# The effects of novel electrical teat dipping on some mastitis parameters in dairy herds

**DOI:** 10.5194/aab-66-141-2023

**Published:** 2023-03-24

**Authors:** Tarik Safak, Ali Risvanli, Oznur Yilmaz, Burak Yuksel, Nevzat Saat, Burak Tanyeri

**Affiliations:** 1 Department of Obstetrics and Gynecology, Faculty of Veterinary Medicine, Kastamonu University, Kastamonu, Türkiye; 2 Department of Obstetrics and Gynecology, Faculty of Veterinary Medicine, Fırat University, Elazığ, Türkiye; 3 Department of Obstetrics and Gynecology, Faculty of Veterinary Medicine, Kyrgyz-Turkish Manas University, Bishkek, Kyrgyzstan; 4 Department of Obstetrics and Gynecology, Faculty of Veterinary Medicine, Siirt University, Siirt, Türkiye; 5 Department of Obstetrics and Gynecology, Faculty of Veterinary Medicine, Balıkesir University, Balıkesir, Türkiye; 6 Department of Airframe and Powerplant Maintenance, Civil Aviation School, Fırat University, Elazığ, Türkiye

## Abstract

Electrical teat dipping (ETD) is a novel, patented method developed by the authors to control mastitis in dairy cows. Here we evaluate the efficacy of ETD in reducing the incidence of clinical mastitis and bulk tank milk somatic cell count (BTMSCC) on three dairy farms over 6 months. ETD was applied for morning and evening milking on three farms, while conventional teat dipping (CTD) was applied on the other three farms. The number of animals and quarters with clinical mastitis and monthly BTMSCC measurements were recorded. We found that the incidence of clinical mastitis was lower on farms using ETD than those using CTD. However, the BTMSCC did not significantly change throughout the study. Based on these findings, we conclude that ETD effectively reduces mastitis rates on dairy farms.

## 1 Introduction

Despite various control methods, mastitis continues to be a major challenge in the dairy industry. One of the key factors contributing to the persistence of mastitis is the prolonged opening of the teat canal after milking, which can last up to 2 h (Borucki et al., 2012; Kuplulu and Vural, 2016). To address this issue, Risvanli et al. (2022) developed and patented electrical teat dipping (ETD), a device that immediately closes the teat canal after milking.

Although teat dipping has been widely recognized as an effective way to prevent mastitis, many farms still struggle to achieve desired reductions in incidence rates (Kuplulu and Vural, 2016). Moreover, teat dipping can lead to problems such as bacterial resistance and residue development (Borucki et al., 2012). The ETD device, which applies 1.8–3 V and 5.5–7.2 A linear current to an antiseptic solution, offers a safe and effective alternative (Risvanli et al., 2022; Yilmaz et al., 2022).

The objective of this study was to evaluate the impact of ETD on clinical mastitis rates and bulk tank milk somatic cell count (BTMSCC) on dairy farms. By applying ETD during morning and evening milking on three farms, and conventional teat dipping (CTD) on three other farms, we could compare the incidence of clinical mastitis and BTMSCC between the two groups over 6 months.

**Table 1:** Evaluation of the results according to the quarters and cows.

		First month		Second month		Third month		Fourth month		Fifth month		Sixth month	
		n	%	n	%	n	%	n	%	n	%	n	%
Cows with mastitis	ETD ( n = 182)	7	3.85	1	0.55	0	0.00	0	0.00	0	0.00	1	0.55
	CTD ( n = 398)	18	4.52	20	4.03	18	4.52	16	4.12	10	2.51	16	4.02
	$\chi^{2}$	0.879		0.015		0.008		0.014		0.031		0.042	
Quarters with mastitis	ETD ( n = 728)	9	1.24	1	0.14	0	0.00	0	0.00	0	0.00	1	0.14
	CTD ( n = 1588)	23	1.45	27	1.70	24	1.51	19	1.20	13	0.82	20	1.26
	$\chi^{2}$	0.830		0.003		0.002		0.007		0.001		0.016	

ETD: electrical teat dipping, CTD: conventional teat dipping.

**Table 2:** Evaluation of bulk tank milk somatic cell counts on farms according to months.

	Bulk tank milk somatic cell count ( $\times 10^{3}$ ) (cell mL ${}^{-1}$ ) ( $\bar{x}\pm s_{\bar{x}}$ )					
	First month	Second month	Third month	Fourth month	Fifth month	Sixth month
ETD	321.70 ± 77.70	216.00 ± 85.40	291.70 ± 57.30	217.00 ± 72.20	210.0 ± 56.80	214.70 ± 64.10
CTD	233.30 ± 59.60	255.30 ± 85.60	198.30 ± 71.00	213.30 ± 70.00	187.00 ± 58.80	221.7 ± 64.10
$\chi^{2}$	0.275	0.827	0.275	0.827	0.513	1.000

ETD: electrical teat dipping, CTD: conventional teat dipping.

## 2 Material and methods

### 2.1 Animals and sampling

This study was conducted in 2021 on six dairy farms in and around Elazığ, Türkiye, after obtaining approval from the Fırat University Animal Experiments Local Ethics Committee (2019/101).

Three farms were selected for ETD (Turkish patent no. 2021 001883B, PCT-TR2021-36) application, while the other three received CTD for 6 months after each morning and evening milking. Mastitis-related data, including the number of cows and quarters with clinical mastitis and BTMSCC, were recorded monthly for all six farms. The BTMSCC was measured using a DeLaval Cell Counter^®^ (Cell Counter DCC; DeLaval, Sweden) device within an hour of milk sample collection (Safak and Risvanli, 2021). Clinical mastitis was defined as mammary glands with local inflammation symptoms (e.g. redness, pain, and swelling) and abnormal milk (Sharun et al., 2021).

### 2.2 Statistical analysis

Statistical analysis was performed using the Mann–Whitney U test to compare the differences between the BTMSCC groups, and the $\chi^{2}$ test was used to compare the differences between the groups for mastitis cases observed in the animals and quarters. Pearson and continuity correction results were considered based on the minimum expected number. The SPSS Statistics 22.0 program (Statistical Package for the Social Sciences for Windows, Chicago, Illinois, USA) was used for these analyses.

## 3 Results

The incidence of clinical mastitis was evaluated for 6 months on all six farms where the application was performed. The observed mastitis cases were lower on the three farms using ETD compared to the previous months in both quarters (first month = 1.24 %, second month = 0.14 %, third month = 0.0 %, fourth month = 0.0 %, fifth month = 0.0 %, and sixth month = 0.14 %) and cows (first month = 3.85 %, second month = 0.55 %, third month = 0.0 %, fourth month = 0.0 %, fifth month = 0.0 %, and sixth month = 0.55 %) (Table 1). On the other hand, the BTMSCC did not differ significantly between the farms where ETD and CTD were applied, as evaluated over 6 months (Table 2).

## 4 Discussion

Teat dipping is an essential component of mastitis prevention control in dairy herds. However, despite its effectiveness, it still has some limitations. In particular, the incidence of mastitis does not decrease to the desired levels in some herds, and the residual problem remains a major challenge (Yanuartono et al., 2020). Teat-dipping solutions, such as chlorinated and iodinated antiseptics, are commonly used in the industry, with iodine concentrations varying from 0.05 % to 3 % (Boddie et al., 2000; Kuplulu and Vural, 2016). However, as the iodine concentration increases, residue problems in milk become more significant. A Canadian study showed that milk in bulk tanks had an iodine content of 54 to 1902 µ g L ${}^{-1}$ (Borucki et al., 2012), underscoring the need for new techniques.

The ETD device, developed and patented by the authors of the presented study, combines CTD application with the electrical field stimulation technique. The device operates with a voltage between 1.8 and 3 V and a current between 5.5 and 7.2 A. Risvanli et al. (2022) reported that the teat canal closed more quickly after ETD application than after CTD, according to ultrasonographic examinations. In our study, we found that clinical mastitis cases were lower on the farms using ETD than those using CTD. However, the BTMSCC did not differ significantly between the two groups over the 6-month study period. We also observed no adverse effects on the animals during or after the study. Although we could not compare our results with a similar ETD device, our findings suggest that ETD performs better than CTD in reducing mastitis rates, and the linear current applied does not pose a problem regarding animal welfare and ethics. Reinemann (2012) reported that exposure to stray voltage levels of 2 to 4 V is considered a mild stressor to some dairy cows, but it does not contribute to increased somatic cell count or incidence of mastitis, or reduced milk yield. Stray voltage is an alternating current, whereas the current applied in ETD is linear.

## 5 Conclusion

In conclusion, based on our results, we conclude that it would be beneficial to expand the use of the device, as we can say that ETD reduces the incidence of mastitis in dairy herds.

## Data Availability

The original data of the paper are available from the corresponding author upon reasonable request.
